# Systems toxicology study reveals reduced impact of heated tobacco product aerosol extract relative to cigarette smoke on premature aging and exacerbation effects in aged aortic cells in vitro

**DOI:** 10.1007/s00204-021-03123-y

**Published:** 2021-07-27

**Authors:** Carine Poussin, Marco van der Toorn, Sophie Scheuner, Romain Piault, Athanasios Kondylis, Rebecca Savioz, Rémi Dulize, Dariusz Peric, Emmanuel Guedj, Fabio Maranzano, Celine Merg, Moran Morelli, Anne-Laure Egesipe, Stéphanie Johne, Shoaib Majeed, Claudius Pak, Thomas Schneider, Walter K. Schlage, Nikolai V. Ivanov, Manuel C. Peitsch, Julia Hoeng

**Affiliations:** 1 PMI R&D, Philip Morris Products S.A., Quai Jeanrenaud 5, CH-2000 Neuchâtel, Switzerland; 2 Biology Consultant, Max-Baermann-Str. 21, 51429 Bergisch Gladbach, Germany; 3 Consultants in Science Sàrl, Biopole, Route de la Corniche 4, 1066 Epalinges, Switzerland

**Keywords:** Heated tobacco product, Modified risk tobacco product, Cigarette, Smooth muscle cells, Vascular diseases, Aging

## Abstract

Aging and smoking are major risk factors for cardiovascular diseases (CVD). Our in vitro study compared, in the context of aging, the effects of the aerosol of Tobacco Heating System 2.2 (THS; an electrically heated tobacco product) and 3R4F reference cigarette smoke (CS) on processes that contribute to vascular pathomechanisms leading to CVD. Young and old human aortic smooth muscle cells (HAoSMC) were exposed to various concentrations of aqueous extracts (AE) from 3R4F CS [0.014–0.22 puffs/mL] or THS aerosol [0.11–1.76 puffs/mL] for 24 h. Key markers were measured by high-content imaging, transcriptomics profiling and multianalyte profiling. In our study, in vitro aging increased senescence, DNA damage, and inflammation and decreased proliferation in the HAoSMCs. At higher concentrations of 3R4F AE, young HAoSMCs behaved similarly to aged cells, while old HAoSMCs showed additional DNA damage and apoptosis effects. At 3R4F AE concentrations with the maximum effect, the THS AE showed no significant effect in young or old HAoSMCs. It required an approximately ten-fold higher concentration of THS AE to induce effects similar to those observed with 3R4F. These effects were independent of nicotine, which did not show a significant effect on HAoSMCs at any tested concentration. Our results show that 3R4F AE accelerates aging in young HAoSMCs and exacerbates the aging effect in old HAoSMCs in vitro, consistent with CS-related contributions to the risk of CVD. Relative to 3R4F AE, the THS AE showed a significantly reduced impact on HAoSMCs, suggesting its lower risk for vascular SMC-associated pathomechanisms leading to CVD.

**Supplementary Information:**

The online version contains supplementary material available at 10.1007/s00204-021-03123-y.

## Introduction

Age, independent of other conventional risk factors, constitutes a major risk factor for the development of cardiovascular diseases (CVD), such as atherosclerosis and abdominal aneurysms (Forsdahl et al. 2009; Kent et al. 2010; Office of the Surgeon General (US) 2010; Messner and Bernhard 2014). Population studies have shown that more than 80% of individuals aged 65 years and above exhibit intracranial carotid artery atherosclerosis (Bos et al. 2012). In the Life Line Screening cohort, 80% of 23,446 subjects with abdominal aortic aneurysm (AAA) were over 65 years old (Kent et al. 2010). Vascular dysfunctions are a common hallmark of many age-related diseases. One characteristic of aging is the accumulation of senescent cells (i.e., cells that irreversibly stop dividing) resulting from a disequilibrium between the accumulation of damaged cells and insufficient repair/clearance of damaged cell components (e.g., defective autophagy) or cells (Lopez-Otin et al. 2013). Aging-triggered senescence (replicative senescence) occurs normally because of the limited number of replication cycles per cell, which is controlled by telomere shortening (Harley et al. 1990; Chi et al. 2019). However, premature cell senescence can occur by environmental or extrinsic insults that induce stressors, such as oxidative stress, inflammation, and DNA damage (Chi et al. 2019).

Vascular smooth muscle cells (VSMC), which are localized in the vascular media, play a key role in maintaining the structure and function (e.g., vasotone) of the vascular wall (Wilson 2011). Senescence of VSMCs induces multiple changes in their responsiveness to vasomodulators, signaling pathways, communication with extracellular matrix (ECM), and phenotype (e.g., contractile to synthetic phenotype; Chi et al. 2019). Moreover, senescent VSMCs develop a senescence-associated secretory phenotype (SASP), characterized by the secretion of pro-inflammatory mediators in the vascular wall. Overall, these changes create a microenvironment that favors chronic vascular inflammation and the development of age-related pathologies (Ovadya and Krizhanovsky 2014; Zhu et al. 2014; Chi et al. 2019). Evidence indicates that SMC senescence is implicated in arterial stiffness, a disorder that contributes to hypertension, and exerts a role in atherosclerotic plaque vulnerability (Wang et al. 2015; Chi et al. 2019; Harman and Jorgensen 2019). In the context of mimicking aging, VSMC senescence can be induced in vitro either by multiple passages (replicative senescence) or by applying a stress, such as doxorubicin or angiotensin II treatment (stress-induced senescence; Chi et al. 2019), which is also used to promote AAA in mice (Lysgaard Poulsen et al. 2016). Aged primary VSMCs can also be harvested directly from old donors and compared with cells from young donors (Ruiz-Torres et al. 1999; Guntani et al. 2011; Thompson et al. 2014; Riches et al. 2018), with the disadvantage that any observed differences may result from the combined effects of differences in genetic background, thus confounding the contribution of aging.

Smoking is recognized as a major risk factor for CVD and has been reported to promote stressors—such as inflammation, oxidative stress, and DNA damage—that contribute to the senescence of VSMCs (Csordas et al. 2008; Bos et al. 2012; Kent et al. 2010; Alsaad et al. 2019; Virani et al. 2020). However, to our knowledge, no study has reported a direct link between cigarette smoke (CS) exposure and induction of VSMC senescence, unlike in the case of other human cell types, such as lung fibroblasts, normal alveolar epithelial cells, endothelial cells, and macrophages (e.g., in atherosclerosis; Tsuji et al. 2004; Farhat et al. 2008; Nyunoya et al. 2006; Favero et al. 2014; Wu et al. 2020). Studies investigating the impact of CS extract or nicotine exposure on VSMCs have reported effects on autophagy, phenotype switching, migration, and proliferation, which are processes implicated in atherosclerosis and plaque stability/instability (Yoshiyama et al. 2011, 2014; Guo et al. 2017; Wang et al. 2019). Some studies provide evidence that smoking may accelerate aging and, subsequently, age-related diseases (Ho et al. 2012; Walters et al. 2014; Vij et al. 2018; Earls et al. 2019; Mamoshina et al. 2019; Yang et al. 2019; Choukrallah et al. 2020).

Tobacco harm reduction, aimed at replacing cigarettes with less harmful products, is a complementary approach to current strategies that aim at smoking prevention and cessation and is intended for smokers who would otherwise continue to smoke. To help achieve this goal, several diverse smoke-free products have been developed, such as heated tobacco products and e-vaping products. However, it is necessary to carefully assess these products through a multi-disciplinary approach to evaluate their potential for reducing toxicant exposure and health risks in comparison with conventional tobacco products (Bolt 2020). Combustion of tobacco releases thousands of toxic chemicals, including acrolein, benzene, and cadmium (Perfetti and Rodgman 2011; Haussmann 2012; Rodgman and Perfetti 2016). In contrast, heating rather than burning tobacco significantly decreases the number and levels of harmful constituents in the aerosol (Werley et al. 2008; Schorp et al. 2012). Tobacco Heating System 2.2 (THS) is a novel heated tobacco product, and analysis of its aerosol has shown an average reduction of more than 90% in the levels of toxicants relative to CS (Schaller et al. 2016). Recent evaluations of the biological impact of THS aerosol have shown its reduced exposure effects relative to the smoke of a reference cigarette on the respiratory and cardiovascular systems in vivo and in vitro (van der Toorn et al. 2015a; Phillips et al. 2016, 2019; Poussin et al. 2016, 2020; Szostak et al. 2020). A 6-month randomized, controlled, 2-arm parallel-group, multicenter, ambulatory Exposure Response Study (ZRHR-ERS-09-US study) in healthy adult smokers who switched from cigarette smoking to THS use (*n*  =  488) or continued to smoke cigarettes (*n*  =  496) in the United States (US) showed favorable changes in eight co-primary endpoints representative of mechanisms including oxidative stress, inflammation, endothelial dysfunction, platelet activation, lipid metabolism, oxygen transport, lung function, and carcinogen exposure (Ludicke et al. 2019).

In the present study, we investigated the biological impact of CS and THS 2.2 aerosol in the form of aqueous extracts (AE) as well as the impact of nicotine in “young” (low passages) and “old/aged” (high passages) primary human aortic vascular smooth muscle cells (HAoSMC) using the replicative senescence approach for aging cells in vitro. Processes that can promote vascular pathomechanisms when dysregulated—including senescence, proliferation, oxidative stress, DNA damage, autophagy, apoptosis, inflammation, and proteolysis—were examined by assays for measuring specific markers (e.g., β-galactosidase, cytokines, chemokines, metalloproteinases, and pH2AX). Broader molecular changes were additionally investigated by profiling the HAoSMC transcriptome. The study allowed us to learn about the changes that occur in HAoSMCs upon in vitro aging at baseline and upon exposure to 3R4F CS AE or nicotine in the context of young and old cells and compare these changes with those caused by exposure to the AE of THS aerosol.

## Materials and methods

### Generation of fresh AEs from 3R4F smoke and THS aerosol

3R4F reference cigarettes were purchased from the University of Kentucky (Lexington, KY, USA; http://www.ca.uky.edu/refcig/), and THS sticks were provided by Philip Morris Products S.A. (Neuchâtel, Switzerland). Prior to AE generation, the 3R4F cigarettes and THS sticks were conditioned for a minimum of 48 h at 22  ±  1 °C and a relative humidity of 60  ±  3%, in accordance with ISO standard 3402. Smoke from 3R4F cigarettes was generated on a 20-port Borgwaldt smoking machine (Hamburg, Germany), and aerosol from THS 2.2 was generated on a 30-port SM2000/P1 smoking machine (PMI, Neuchatel, Switzerland) in accordance with the Health Canada Intense (HCI) smoking protocol (55-mL puff volume; 2-s puff duration; 2-min^−1^ puff frequency; and 100% blocking of ventilation holes for 3R4F) (Health Canada 1999). AEs were generated by bubbling aerosol or smoke through C-22262 smooth muscle cell basal medium 2 (PromoCell, Heidelberg, Germany) on ice (3R4F—6 cigarettes/36 mL and 64.4 mean total puffs, resulting in a stock solution concentration of 1.79 puffs/mL; THS—10 sticks/40 mL medium and 120 total puffs, resulting in a stock solution concentration of 3 puffs/mL). All AE solutions (except those used for the proliferation and senescence assays) were further diluted in C-22262 basal medium to obtain final concentrations of 0.014–0.22 puffs/mL for 3R4F and 0.11–1.76 puffs/mL for THS. The AEs used for the proliferation and senescence assays were diluted in C-22062 smooth muscle cell growth medium 2 (PromoCell—Basal medium supplemented with C-39267 Supplement mix).

### Determination of nicotine and carbonyl content in AEs

To monitor batch consistency, the concentrations of nicotine and eight carbonyls in whole smoke/aerosol AEs were determined. For analysis of nicotine, a 100 µL was taken from each AE. Later sample of AE was transferred to a vial containing 900 µL *n*-butyl acetate and 0.1% trimethylamine, with isoquinoline as the internal standard. Nicotine analysis was performed by gas chromatography (GC) with flame ionization detection (GC-FID) as previously described (van der Toorn et al. 2018). Analysis of the eight carbonyls was performed by liquid chromatography–electrospray ionization tandem mass spectrometry (LC-ESI–MS/MS) as described by Van der Toorn et al. (van der Toorn et al. 2015b). Briefly, the carbonyl compounds were derivatized and quenched to stabilize them. A 500 µL aerosol-derivatized sample was introduced in an LC–MC glass vial containing the internal standard working solution. The carbonyl compound concentrations were calculated using an external calibration curve. All results are expressed as µg per item trapped.

### Cell culture and aging in vitro

Primary HAoSMCs (C-12533; Lot 399Z005) were obtained from PromoCell (Heidelberg, Germany) and cultured in C-22062 growth medium (PromoCell) in accordance with the recommendations of the distributor. The cells were grown in 75-cm^2^ collagen I-coated culture flasks (VWR, Dietikon, Switzerland) at 37 ℃ in an atmosphere with 5% CO_2_ until 75–80% confluence. Cells in suspension were counted with a CASY® counter (Roche Diagnostics International Ltd., Mannheim, Germany). HAoSMCs cultured and frozen at passages 3 and 4 were considered as young cells. The HAoSMCs were aged in vitro by replicative senescence (Nakano-Kurimoto et al. 2009; Bielak-Zmijewska et al. 2014). Thus, HAoSMCs grown and frozen in liquid nitrogen at passages 12 and 13, at which point proliferation had decreased, were considered as old cells. For all experiments, young and old cells were seeded in the same plate before exposure.

## Cell exposure

HAoSMCs at passage 3–4 (young cells) were seeded in black collagen I-coated, clear-bottom 96-well tissue culture plates (BD, Allschwil, Switzerland) at a density of 12,000 cells/well (young cells used for the proliferation and senescence assays were seeded at 5000 cells/well) in C-22062 growth medium. HAoSMCs at later passages (12–13; old cells) were seeded at a lower density of 8000 cells/well, because the old cells were much larger than the young cells (old cells used for the proliferation and senescence assays were seeded at 5000 cells/well). A day after seeding, the growth medium was replaced with starvation medium (C-22262 basal medium supplemented with 0.1% bovine serum albumin), except in the proliferation and senescence assays, where the cells were maintained in growth medium. At the same time, the cells were treated (in duplicate) with nicotine (Sigma-Aldrich Chemie GmbH, Buchs, Switzerland; 99% liquid; 5 mL) at 0.15, 0.5, 1.5, 5, and 15 µM, AE from 3R4F smoke at 0.014, 0.028, 0.055, 0.11, and 0.22 puffs/mL, or AE from THS aerosol at 0.11, 0.22, 0.44, 0.88, and 1.76 puffs/mL and incubated for 24 h. Regarding nicotine, the lowest test concentration in this study was 0.15 µM, chosen for biological relevance to be within the range of nicotine concentrations (0.07–0.23 µM) observed in the plasma of cigarettes smokers (Russell et al. 1981; D’Ruiz et al. 2015; Brossard et al. 2017). The test concentration ranges for 3R4F and THS AEs were based on prior concentration range-finding experiments, where these concentrations were shown to induce a satisfactory range of response for the various in vitro endpoints without causing significant cytotoxicity. The two highest concentrations used for 3R4F AE (i.e., 0.11 and 0.22 puffs/mL) were used as the starting concentrations for THS AE to have bridging concentrations between the two products.

Cells were also exposed to control medium (i.e., starvation medium for all assays except the senescence and proliferation assays, for which growth medium was used as the control medium) as well as to positive controls and their corresponding vehicles (as vehicle controls) in two, three or four replicate wells. Two positive controls were used for the oxidative stress assay: 1000 µM tacrine and 300 µM rotenone in 0.1% DMSO (v/v; dimethyl sulfoxide). Chlorambucil and mitomycin, both at 100 µM in 0.1% DMSO (v/v), served as positive controls for the DNA damage assay. No positive control was used for the autophagy assay; the cells were treated with 50 µM chloroquine, used as a blocker of the autophagosome–lysosome fusion enabling the accumulation in the autophagosome of microtubule-associated proteins 1A/1B light-chain 3B (LC3B) protein measured in the assay. For the proliferation and senescence assays, 100 µM chlorambucil and 30 µM aphidicolin in 0.1% DMSO (v/v) were used as positive controls. For the apoptosis assay, 2 µM staurosporine in 0.1% DMSO (v/v) served as the positive control. Tumor necrosis factor α (TNFα) in 0.1% saline and PMA (phorbol 12-myristate 13-acetate in 0.001% DMSO (v/v)), each at 10 ng/mL, were the positive controls of inflammatory response for the proteomics and transcriptomics assays.

### High-content screening

A high-content screening (HCS) approach was used to quantify parameters relevant to the cell cycle (proliferation), senescence, DNA damage, autophagy, apoptosis, and oxidative stress (Marescotti et al. 2016). After the 24-h exposure period, the cells were stained with specific reagents for each endpoint: Hoechst 33342 was used to quantify senescence and proliferation; a specific antibody targeting pH2AX was used to assess DNA damage; autophagy was assessed with a commercially available kit (LC3B Antibody Kit for Autophagy—Catalog No. L10382; Invitrogen, Eugene, OR) based on an antibody targeting L3CB; apoptosis was evaluated by measuring caspase 3/7 intensity; and dihydroethidium (DHE) fluorescence was used to quantify oxidative stress. After HAoSMC staining, fluorescence data were acquired with a Thermo Fisher Cellomics® ArrayScanVTI High Content Screening Reader (Thermo Fisher Scientific Inc., Waltham, MA, USA) and vHCS view software (Thermo Fisher Scientific Inc.). Twenty fields were imaged per well using a 10×wide-field objective.

### Multianalyte profiling

Supernatants from HAoSMCs were sampled for quantification of proteases [matrix metalloproteinase (MMP)-1, MMP-2, MMP-7, MMP-9, and MMP-10] and cytokines/chemokines [epidermal growth factor (EGF), interferon gamma (IFN)γ, interleukin (IL)-1α, IL-1β, IL-6, IL-8, IL-10, monocyte chemoattractant protein (MCP)-1, RANTES, and tumor necrosis factor (TNF)α]. The levels of these analytes were measured with the Luminex® xMAP® (Luminex Corporation, Austin, TX, USA) technology using the commercially available kits MILLIPLEX® MAP Human MMP Panel 2 (Catalog no. HMMP2MAG-55 K-05; Merck KGaA, Darmstadt, Germany) and MILLIPLEX® MAP Human Cytokine/Chemokine (Catalog No. HCYTOMAG-60 K-10C; Merck KGaA). The samples were used undiluted or diluted 50- or 250-fold. They were then assayed in duplicate in accordance with manufacturer’s protocol, and the plates were read with a Luminex® FLEXMAP 3D® instrument equipped with the xPONENT® software (Version 4.2, Luminex Corp). The concentrations of the analytes (pg/mL) were determined by the instrument after back-fitting the median fluorescence intensities using a five-parameter logistic curve.

### Transcriptomics

HAoSMCs were lysed in 233 μL RLT lysis buffer (Qiagen, Hilden, Germany), and the lysates were frozen at − 80 ℃ for RNA extraction and profiling using the GeneChip® Human Genome U133 Plus 2.0 array (Thermo Fisher). Before processing, the control and treatment cell lysate samples (from three or four independent experiments) were randomized using the plate factor as a blocking factor to avoid any confounding effect.

RNA was extracted with a Qiagen Qiacube robot (batch of 12 samples) using the RNeasy Micro kit (Qiagen, #74004, Hilden, Germany) and quantified using Nanodrop ND-1000 (Thermo Fisher). RNA quality was determined using an Agilent 2100 Bioanalyzer with the Agilent RNA 6000 Pico Kit (Agilent Technologies, #5067-1513, Santa Clara, CA, USA). The RNA samples from HAoSMCs had concentrations ranging from 2.5 to 23.4 ng/µL and RIN (RNA integrity number) ranging from 7.2 to 10 (average, 9.6).

mRNAs from HAoSMCs were all processed in a 96-well plate (same batch) and required 2.0 ng of total RNA; this process was performed with a fully automated Biomek FxP robot (Beckman Coulter, Indianapolis, USA) and the ThermoFisher GeneChip™ 3′ IVT Pico Kit (Cat. #902789 ThermoFisher, Waltham, MA, USA). The mRNAs (2.0 ng) were converted to complementary RNA (cRNA) and amplified by low-cycle PCR using T7 in vitro transcription (IVT) technology. The cRNA was converted to biotinylated double-stranded cDNA (ds cDNA) hybridization targets, which were then fragmented and biotinylated. Hybridization was performed overnight (16 h) at 60 rpm in a 45 ℃ GeneChip® Hybridization Oven 645 (Thermo Fisher) on a Genechip Human Genome U133 Plus 2.0 Array (#900467; Thermo Fisher), which simultaneously probes the expression of thousands of genes. The sequences from which these probe sets were derived were selected from GenBank, dbEST, and RefSeq, with complete coverage of the Human Genome U133 set as well as 6500 additional genes for analysis of over 47,000 transcripts. The arrays were washed and stained on a GeneChip® Fluidics Station FS450 DX (Thermo Fisher) using the Affymetrix® GeneChip® Command Console® Software (AGCC software v 3.2; protocol FS450_0001) and scanned on GeneChip™ Scanner 3000 7G to generate ‘.dat’ files, which were automatically converted to CEL files.

### Computational transcriptomics data analysis

#### Data processing and pairwise comparison

Probe intensities corresponding to the raw data (CEL files are available on the INTERVALS platform at https://doi.org/10.26126/intervals.x3xkne.1) were summarized using the revised Entrez-based probe annotation of Dai (HGU133Plus2_Hs_ENTREZG cdf v16.0.0) (Dai et al. 2005) and normalized by frozen robust microarray analysis (fRMA; McCall et al. 2010). The normalization vector HGU133Plus2_fRMAvecs version 1.3.0 was used with the R package fRMA version 1.18.0. Quality control was performed with the affyPLM package version 1.42.0 (Bolstad 2005). Differential gene expression analysis was performed using the Bioconductor Limma R package version 3.22.4 (Smyth 2004). Pairwise comparisons at the gene level, called “systems response profiles” (SRP), were computed by comparing the gene expression levels of (i) old and young HAoSMCs in control medium (named “C-Oy”; aging effect at baseline); (ii) each 3R4F AE or THS AE concentration in young and old HAoSMC cultures with the respective vehicle control (named “3R4F/THS-CONCENTRATION-Y/O”; product effect in young or old HAoSMCs); and (iii) each 3R4F AE or THS AE concentration in old HAoSMC cultures with the vehicle control in young HAoSMC cultures (named “3R4F/THS-CONCENTRATION-Oy”; combined aging and product effect). Genes with a false discovery rate (FDR; corresponding to *p* value adjustment by the Benjamini–Hochberg method) below 0.05 were considered differentially expressed genes (DEG; Benjamini and Hochberg 1995). To support biological interpretation, SRPs including all genes (∼18,000) were further leveraged in downstream computational analyses (gene set enrichment, principal component, and network perturbation amplitude analyses). Most R packages used for the data analysis were included in Bioconductor 3.0 version. The R version used was 3.1.2 on R-Studio Server Pro.

#### Network perturbation amplitude analysis

Network perturbation amplitude (NPA) analysis has been performed to quantify the level of perturbations of causal networks representative of main processes including cell fate, cell proliferation, cell stress, tissue repair and angiogenesis and inflammation (Online reference 1).

#### Gene set enrichment analysis of old vs. young SRPs at baseline

Gene set enrichment analysis was conducted to identify biological pathways/processes associated with old vs. young HAoSMC SRPs at baseline. Genes were ranked by their *t *values, and gene resampling (Q1) was performed to generate the null hypothesis distribution and compute the significance associated with each gene set (Ackermann and Strimmer 2009). Two collections of gene sets representative of pathways and biological processes (MSigDB C2-CP and a set of genes included in the RT2 profiler PCR arrays from Qiagen were extracted and also used as a source of gene sets representative of pathways) were used as a priori knowledge.

#### Principal component analysis (PCA) and pathway over-representation analysis

Principal component analyses were performed using the fold-change matrix and various subsets of SRPs to identify the sources of variations corresponding to the main biological effects including cell aging, impact of the highest vs. lower concentrations of product, concentration-dependent product effect, and the combined effects of aging and concentration-dependent product exposure (Online reference 2). For each PCA, genes that contributed the most to the separation of SRPs in the PC1–PC2 subspaces for an effect of interest were considered as “driver genes” and leveraged as prototypic expression patterns to identify associated underlying pathways and biological processes. Thereby, genes whose expression changes correlated the most with each of the driver gene expression patterns were selected on the basis of their Pearson correlation coefficient being  >  0.65 and the associated *p* value being  <  0.05. An over-representation analysis was performed with selected genes and using two independent collections of gene sets representative of pathways and biological processes described in the previous section.

### Statistical analyses

In general, the data were reported as means and standard errors of the mean (SEM), or standard deviations (SD; e.g., carbonyls), where appropriate. Means were calculated from *N* independent experiments (specified for each endpoint in figures and/or figure legends), each experiment consisting of a median of 2–3 replicate wells (*n*). The measured treatment responses were expressed as the logarithm to base 2 (log_2_) of fold changes with respect to the relevant control. Statistical analyses included (1) comparison of each treatment condition with its respective control; (2) comparison of the treatment condition between young and old cells at common concentrations; and (3) comparison between the THS AE and 3R4F AE treatment conditions at common concentrations. All comparisons were performed using the t test procedure for independent or paired samples, where appropriate. In general, the data were standardized against the control medium in young cells. A significance level of 0.05 was set for all analyses. No imputation for missing values was performed. Values below the lower limit of quantification (LLoQ) or above the upper limit of quantification (ULoQ) were used as is in the analyses. If the measurement had no value and was denoted by “  <  LLoQ” or “  >  ULoQ”, it was replaced by LLoQ/2 or 1.1*ULoQ, respectively. To compare the impact of increasing concentrations of 3R4F and THS AEs on progression of the phenotype of the young (passage 3–4) cells towards that of the old (passage 12–13) cells, the differences in proliferation, senescence, DNA damage, autophagy, apoptosis, and oxidative stress between young and old cells (relative to old cells) were each plotted against the log_10_ of the AE concentrations separately for 3R4F and THS. A relative difference of 0% in, for example, DNA damage in cells exposed to 3R4F AE at a concentration of 0.11 puffs/mL would indicate that young cells exhibit the same amount of damage as old cells at the same concentration. Where a concentration-dependent response was observable for a given endpoint–product combination, a non-linear model that could represent the observed relationship was fitted to the data (Mitscherlich for senescence, DNA damage, autophagy, and oxidative stress; Hill for proliferation). The estimated curve was then used to predict (via back-fitting) the concentration of AE at which a 0% relative difference would occur. Because no a priori reason exists for a dissimilar relative difference between 3R4F and THS when the concentration of AE is 0 puffs/mL, the predicted concentrations for both products on the basis of Mitscherlich’s model were derived from two models with the same intercept value. Non-linear model estimates and predicted AE concentrations were used in a purely descriptive manner, and no attempt was made to assess the precision of the obtained concentrations.

All statistical analyses were performed using R-3.2.2 on R-Studio Server Pro.

## Results

### Increasing HAoSMC passage promoted senescence, reduced proliferation, and activated and repressed expression of genes involved in inflammation and the matrisome, respectively

**Fig. 1:**
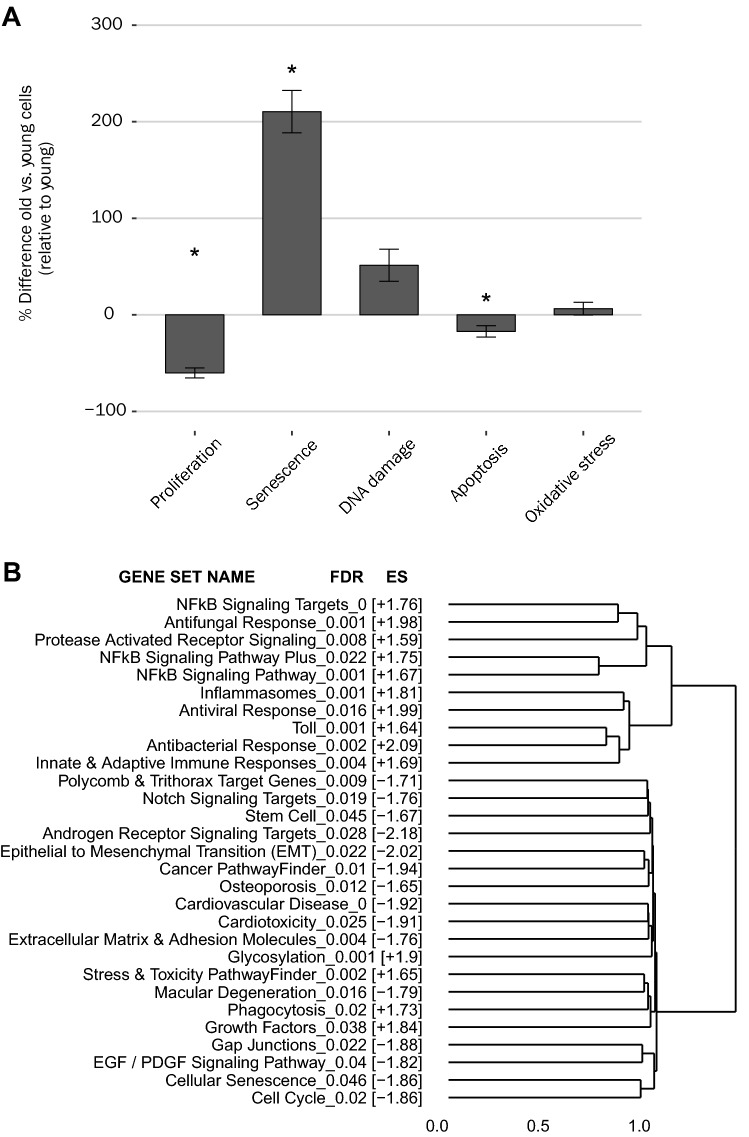
Impact of low (“young”) and high (“old”) cell culture passages in HAoSMCs, a process used for mimicking cell aging in vitro. **A** Comparison of baseline responses of old and young HAoSMCs (treated with control medium) with regard to processes involved in cell stress, damage, and fate. Bars represent means  ±  standard error of the mean from *N*  =  6–7 independent experiments including n  =  3 replicates within each plate. *p  <  0.05 (*t *test young vs. old cells). **B** Gene set enrichment analysis revealed the most significantly [FDR associated with enrichment score (ES)  <  0.05] enriched pathways and diseases associated with up- (+ ES) and downregulated (− ES) gene expression changes in old HAoSMCs relative to young HAoSMCs. Hierarchical clustering (agglomerative method  =  ward.D2) enabled to group gene sets based on their similarities (1-Jaccard index) for leading edge genes contributing to ES. *HAoSMCs* human aortic smooth muscle cells; *ECM* extracellular matrix; *FDR* false discovery rate; *ES* enrichment score

We simulated the process of aging in vitro by increasing the number of primary HAoSMC passages until the cells showed a proliferation slowdown as previously reported (Nakano-Kurimoto et al. 2009; Bielak-Zmijewska et al. 2014). We investigated and compared the functional and molecular changes that occurred at baseline between young and old HAoSMCs (passages 4–5 and 12–13), respectively. At baseline, old cells displayed a significantly different phenotype from young cells with regard to the investigated mechanisms, with lower proliferation and greater levels of markers of senescence, DNA damage, and autophagy than the young cells (Fig. 1A). The baseline levels of apoptosis and oxidative stress tended to be similar in both cell types.

The transcriptome was analyzed in old and young cells in control medium to examine expression changes at the mRNA level. SRPs, also termed contrasts, were computed by comparing DEGs in old and young cells by linear modeling (blocking by “Plate” factor); these are represented in the volcano plots shown in Online resource 3A. Although the same batches of young and old cells were used, the tests with 3R4F (set of experiments a) and THS (set of experiments b) AE were conducted independently in different plates. Therefore, to verify that the cellular response to the aging effect was biologically similar, we compared the SRPs at baseline for experiment sets a (C-Oy. a SRP) and b (C-Oy. b SRP). Genes with associated adjusted *p* values (FDR)  <  0.05 were identified as DEGs (colored in the plots). A significant correlation was observed between the SRPs of experiment sets a and b, with a Pearson correlation coefficient of 0.92 for commonly differentially expressed genes (1264 genes; Online resource 3B). Then, a gene set enrichment analysis was performed by ranking genes of the C-Oy SRP by its t value. Two gene set collections were used as a priori biological knowledge to identify pathways/biological processes that were regulated upon HAoSMC aging in vitro. Overall, the analysis highlighted significant changes in pathways/processes involved in inflammation and immune system activation on the most significantly upregulated side of the ranked gene list (positive ES) and in the matrisome and cell cycle on the most significantly downregulated side (negative ES) of the ranked gene list (Fig. 1B; Online resource 4). Gene sets were clustered by the similarity they shared regarding leading edge genes.

### Exposure to 3R4F AE caused premature aging of young HAoSMCs and exacerbated aging effects in old HAoSMCs—reduced impact of THS AE

**Fig. 2:**
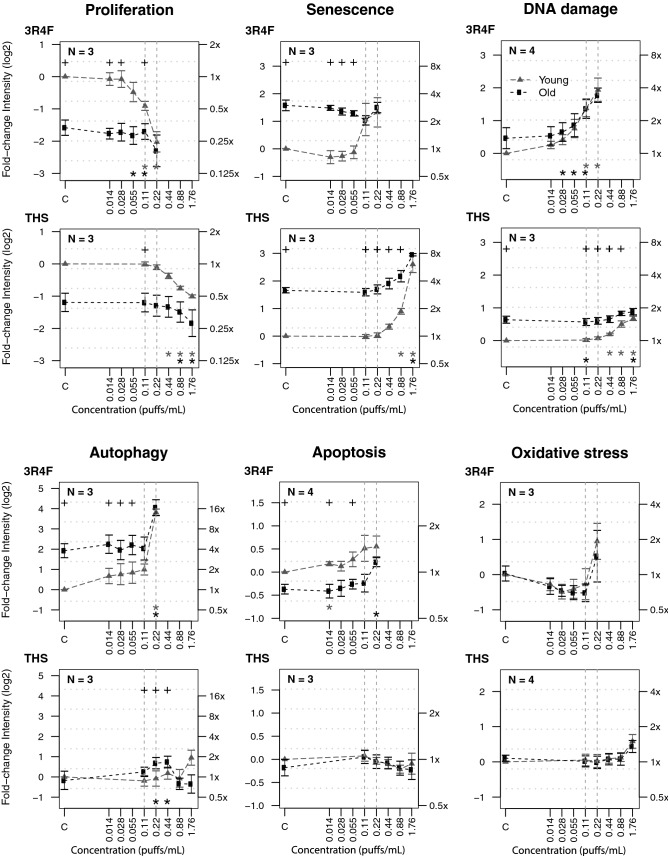
Concentration-dependent effects of 3R4F and THS AEs on processes involved in cell stress, damage, and fate in young and old HAoSMCs. The responses of young (filled gray triangles) and old cells (filled black squares) to various concentrations (log_10_ scale) of AEs is plotted as the fold change in intensity (log_2_ and original scales on left and right y-axes, respectively) relative to the control (C) of young cells. Statistical analyses: black crosses at the top of the plot indicate statistical differences in pairwise comparisons between young and old cells at a significance level of 0.05. Stars at the bottom of the plot indicate whether the cell response for each treatment condition is statistically different from that in the respective control for young cells (gray stars) and old cells (black stars) separately (*p  <  0.05). Points reflect the mean of *N* independent experiments including n  =  2 replicates within plate. Error bars correspond to their standard errors of the mean. *AE* aqueous extract; *C* control; *THS* tobacco heating system

We investigated the impact of exposure of young and old HAoSMCs to 3R4F CS and THS aerosol in the form of AEs. The concentrations of eight carbonyls were determined by LC–ESI–MS/MS to monitor the batch reproducibility of freshly generated AEs from 3R4F CS and THS aerosol (Online resource 5). At matching concentrations of nicotine, the concentrations of carbonyls showed an average decrease of 86% in THS AE relative to 3R4F AE. To determine the product effect on top of the aging effect and for comparison with young cells, the responses in old cells were calculated relative to those in young cells in control medium (Fig. 2). When old cells were treated with increasing concentrations of 3R4F AE, their proliferation and senescence states remained stable. This observation was supported by the fact that, at baseline (as shown in Fig. 1), the old cells were probably already close to minimal proliferative and maximal senescence states that could not be altered much further by 3R4F AE treatment. However, processes including DNA damage and apoptosis increased in a 3R4F AE concentration-dependent manner, whereas the markers of autophagy and oxidative stress tended to increase only at the highest 3R4F AE concentration of 0.22 puffs/mL.

In comparison with old cells, young HAoSMCs responded to 3R4F AE with a larger magnitude. The cells consistently proliferated less, became increasingly senescent, and displayed increased levels of markers of DNA damage, autophagy, apoptosis, and oxidative stress when treated with increasing concentrations of 3R4F AE. At the highest concentration of 0.22 puffs/mL, the difference from the level observed in control medium reached statistical significance (*p*  <  0.05) for proliferation, DNA damage, and autophagy (Fig. 2). The response levels of the young cells reached those of the old cells with increased concentrations of 3R4F AE (Fig. 2).

When assessing the impact of AE exposure on young and old HAoSMCs, no significant effect was measured with THS AE at a concentration of 0.22 puffs/mL, where a maximum effect was seen with the same concentration of 3R4F AE (Fig. 2). A similar pattern of responses to that promoted by 3R4F AE was observed only when the THS AE concentrations were increased by a factor 10, approximately, with 1.76 puffs/mL being the maximum concentration of THS AE (Fig. 2). Similar to the response observed with 3R4F AE, the response level of young cells reached that of old cells with increased concentrations of THS AE, although only at tenfold higher concentrations than that of 3R4F AE (Fig. 2).

**Fig. 3:**
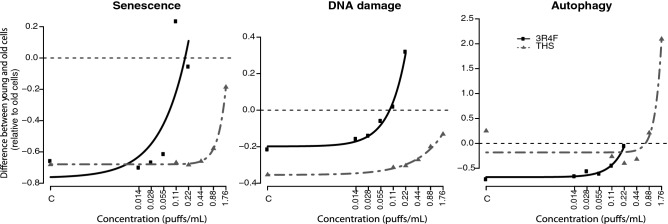
Curve fitting-based extrapolation of concentrations at which young cells reached the response levels of old cells for processes involved in cell stress, damage, and fate. Fitted curves of differences in senescence, DNA damage, and autophagy between young and old cells relative to old cells plotted against product concentration. The Mitscherlich model was used to fit the curves for the three endpoints. Table 1 shows predicted concentrations for 3R4F and THS AE at which young cells reached the response levels of old cells for processes involved in cell stress, damage, and fate. *C* control; *THS* tobacco heating system

To investigate the “aging” effect in young cells exposed to the test AEs in a more quantitative manner, the differences between young and old cells (relative to old cells) were plotted against 3R4F or THS AE concentrations for each endpoint (Fig. 3). Curves were fitted to the data using various models. A satisfactory model could be fitted on the data of both treatments for senescence, DNA damage, and autophagy (Fig. 3). By inverse prediction from the fitted models, the extrapolated concentrations of 3R4F and THS AEs at which the response levels observed in young cells reached those in old cells were extrapolated and compared between the products (Fig. 3; Table 1). The concentrations of 3R4F AE at which young cells were predicted to reach the stages of proliferation, senescence, DNA damage, and autophagy observed in old cells were predicted to be 0.84, 0.16, 0.13, and 0.11 puffs/mL, respectively. The extrapolated concentrations for THS AE relative to 3R4F AE were approximately 12.8 (2.05 puffs/mL)-, 20.6 (2.68 puffs/mL)-, and 7.3 (0.80 puffs/mL)-fold higher for senescence, DNA damage, and autophagy, respectively; a similar comparison could not be made for proliferation (Table 1).

**Table 1:** Prediction of concentrations for 3R4F and THS AE at which young cells reached the response levels of old cells for processes involved in cell stress, damage, and fate

Endpoint	Model	3R4F AE	THS 2.2 AE	Intercept	Ratio
		Predicted concentration (puffs/mL)			THS/3R4F
Proliferation	Hill	> 0.22 (0.84)	n/a^a^	n/a	n/a
Senescence	Mitscherlich	0.16^b^	> 1.76 (2.05)	− 0.70	12.8
DNA damage	Mitscherlich	0.13	> 1.76 (2.68)	− 0.30	20.6
Autophagy	Mitscherlich	0.11	0.80^b^	− 0.25	7.3
Apoptosis	n/a (no model could be fitted)	n/a	n/a	n/a	n/a
Oxidative stress	Mitscherlich^c^	n/a	n/a	n/a	n/a

The concentration at which the difference between old and young cells reached zero was calculated by inverse prediction. The indicated models were fitted using product concentrations on the log_10_ scale, and the intercept was set at the same value for both products (value chosen arbitrarily between the intercept of the 3R4F and THS curves). Predicted concentrations that were out of the range of the tested concentrations are indicated as “ > ” to the maximum product AE concentration tested, with the predicted exact concentration in brackets. For some endpoints, the curve fitting was not applicable (n/a)

*n/a* non-applicable; *THS* tobacco heating system

^a^The difference between young and old cells did not reach 0 according to estimated parameter values

^b^None of the model parameter estimates were statistically significant at an α-level of 0.05; the model was, therefore, not fully reliable

^c^A model could be fitted; however, it was not reliable, as there was no significant difference between young and old cells at any concentration for either product

### In young and old HAoSMCs, 3R4F AE exposure perturbed and exacerbated, respectively, similar biological processes to those observed with aging at baseline-reduced impact of THS AE

After investigating the effect of aging at baseline (Fig. 1), the effect of product exposure on gene expression was analyzed in young and old cells. SRPs were computed by comparing each product–concentration exposure condition in young and old cells with its respective young and old cell control. Graphical representation of SRPs in volcano plots revealed a concentration-dependent increase in the fold changes (x-axis) and significance levels (y-axis) of the effects of 3R4F and THS AEs in young and old cells (Online resource 6). Network perturbation amplitude analysis using transcriptomics data confirmed significant perturbation of causal networks associated with cell fate, such as senescence, cell proliferation, cell stress, inflammation and tissue repair and angiogenesis, when comparing old versus young cells, and when investigating concentration-dependent effects of product AEs (Online resource 7). For most product concentrations, the effect was more pronounced in young cells than in old cells, confirming the results seen in the HCS assays. At 0.22 puffs/mL, where 3R4F AE showed a maximum effect, THS AE exposure produced an extremely low signal (blue rectangle highlights in Online resource 6 and Online resource 7). In both young and old cells, it was necessary to increase the concentration of THS AE by a factor of approximately 10 to observe a similar volcano plot to that obtained with 3R4F AE (Online resource 6; Online resource 7).

**Fig. 4:**
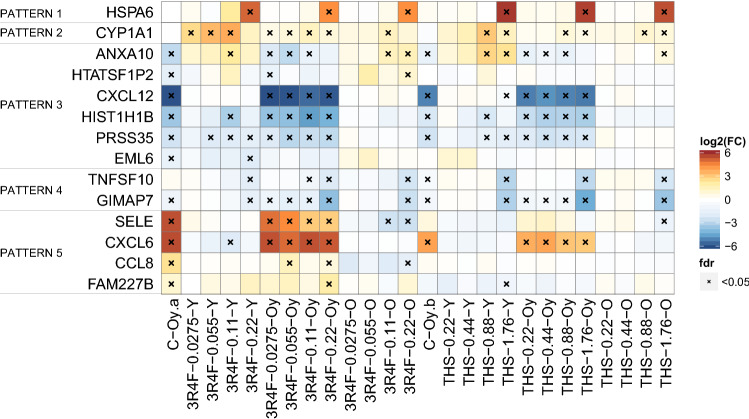
Expression change patterns of driver genes for the main effects revealed by PCA. PCA enabled us to determine the main sources of effects in the FC expression dataset and extract the genes, namely driver genes, that contributed the most to the separation of SRPs for those effects (Online resource 2). Heatmap of the FC expression matrix of driver genes representative of gene expression change behaviors associated with the main sources of effects, such as cell aging, high vs. low concentrations of product, concentration-dependent product exposure, and combined impact of aging and concentration-dependent product exposure. *FC* fold change; *O* old; *Oy* old relative to young; *PCA* principal component analysis; *SRPs* systems response profiles; *Y* young

From the results of PCA and the subsequent over-representation analysis for identifying biological processes/pathways associated with the main biological effects, the most over-represented pathways/biological processes (adjusted *p* value  <  0.0005) associated with the different driver gene patterns (Fig. 4; Online resource 2) are summarized in Table 2 (full list in Online resource 8). In summary, high AE concentrations of the products (0.22 puffs/mL 3R4F AE and 1.76 puffs/mL THS AE) triggered toxic effects in HAoSMCs, with activation of the unfolded protein response (heat shock proteins and chaperones) and cell cycle in an attempt to rescue cells from severe damage (associated with pattern 1 in Table 2). At lower concentrations of the AEs, HAoSMCs activated a concentration-dependent cell stress response to detoxify the xenobiotic-containing milieu (associated with pattern 2 in Table 2). Upon cell aging in vitro, the HAoSMCs underwent gene expression changes, on the one hand, impacting the synthesis of molecules of the matrisome structure and interactions, cell cycle, Notch signaling, and wound healing (associated with pattern 3 in Table 2) and, on the other hand, triggering inflammatory processes, angiogenesis, and Wnt signaling (associated with pattern 5 in Table 2).

**Table 2:** Pathways and biological processes associated with genes correlated with the expression patterns of the driver genes shown in Fig. 4

Driver genes cluster	Most over-represented pathways/diseases—summary
Main effect	(Adjusted p value < 0.0005)
Pattern 1—HSPA6 Low and high product concentration effect	Heat shock proteins and chaperones
	Unfolded protein response
	Cellular stress responses (toxicity, osmotic)
	TGF signaling
	Cell cycle (↑ at high product concentrations)
Pattern 2—CYP1A1 Concentration-dependent product effect	Phase I xenobiotic metabolism^a^
Pattern 3—ANXA10, CXCL12, HTATSF1P2, HIST1H1B, PRSS35, EML6 Cell aging and concentration-dependent product effect	Matrisome (ECM glycoproteins, collagens, integrins, focal adhesion, syndecan-1)
	Cell cycle (↓ by aging and at low product concentrations)
	Notch signaling
	Wound healing
	Epithelial to mesenchymal transition
	Cardiovascular disease
Pattern 4—TNFSF10, GIMAP7 Low and high product concentration effect	Metabolism of lipids (steroid/cholesterol)
Pattern 5—SELE, CCL8, CXCL6, FAM227B Cell aging effect	NFκB signaling
	Cell adhesion molecules
	Lysosome
	Immune system (innate and adaptive), inflammatory response (cytokines and chemokines, protease-activated receptor signaling)
	Inflammasomes
	Angiogenesis
	Wound healing
	Atherosclerosis
	Wnt signaling

Detailed information on PCAs conducted with different subsets of SRPs to unravel the main sources of effects is provided in Online resource 2. All results are provided in Online resource 8

^a^Not statistically significant at the level of FDR < 0.05

**Fig. 5:**
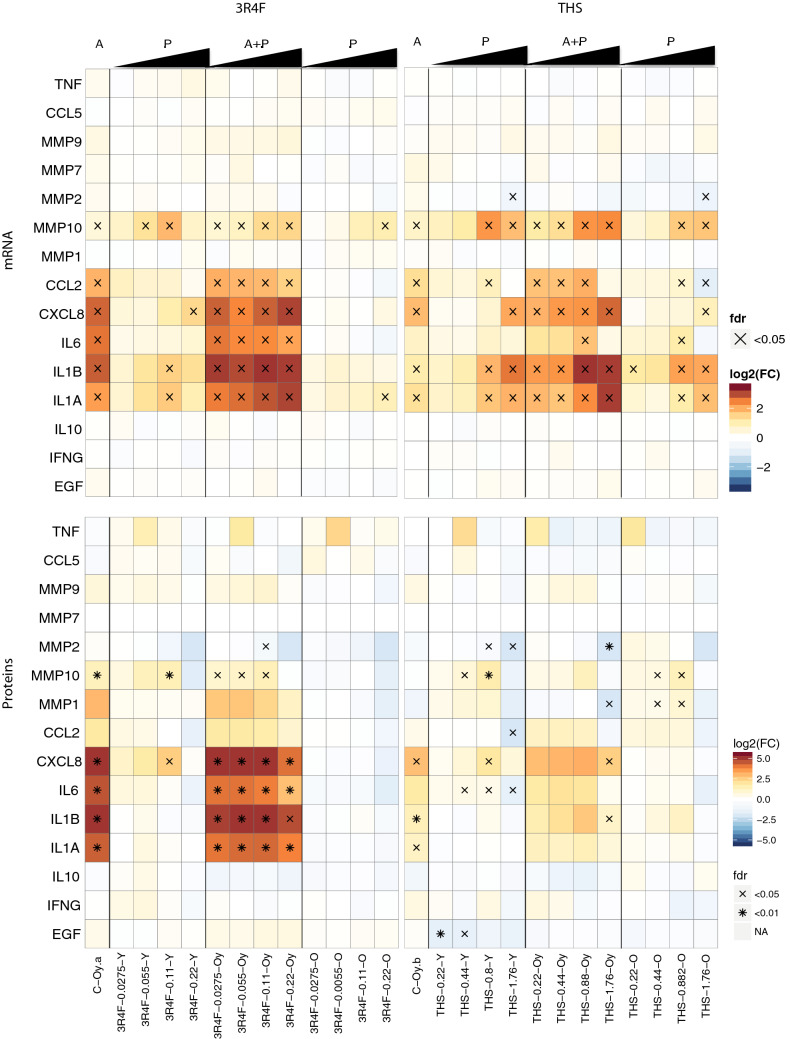
Concentration-dependent effects of 3R4F and THS AE on inflammation and proteolysis markers in young and old HAoSMCs. Gene expression changes are shown in the upper panel. Protein expression changes measured in the cell supernatants are shown in the lower panel. The changes in mRNA expression and protein abundances are expressed as log_2_ fold changes. Adjusted *p* value: FDR; *N*  =  3–4 independent experiments (sets “a” and “b”). “A” represents the aging effect—the responses of old cells in control medium were normalized against those of young cells in control medium (C-Oy). “P” represents the product effect—the responses of young (Y) and old cells (O) were separately normalized against those of their respective controls. “A  +  P” represents the combined effect of aging and the product—the responses of old cells were normalized against those of young cells (Oy). *A* aging; *P* product; *FC* fold change; *O* old; *Oy* old relative to young; *Y* young

The production of inflammatory mediators triggered by the aging process (i.e., senescence-associated secretory phenotype) through cell replication in vitro was confirmed by the presence of increased levels of cytokines including CXCL8, IL-6, IL-1β, and IL-1α and the proteolysis marker MMP-10 in the supernatants of old HAoSMCs relative to young HAoSMCs (Fig. 5). Similar patterns of expression and abundance changes were observed at the mRNA and protein levels, respectively. The exposure of young cells to AE promoted a concentration-dependent release of inflammatory mediators and additional effects to the effect of aging in old cells, observed in a more pronounced manner in the mRNA heatmap (Fig. 5).

Overall, the results show that the exposure of young HAoSMCs to product AEs triggered concentration-dependent molecular changes similar to those promoted by in vitro aging. Old HAoSMCs also showed concentration-dependent additional changes upon AE exposure, although to a lesser extent than the young cells, because the old cells were already overwhelmed by the changes induced by the aging process in vitro. At the concentrations of 3R4F AE at which maximum effects were measured, THS AE showed no significant effect on gene expression changes or associated biological processes or pathways. It was necessary to increase the concentration of THS AE by approximatively 8–10 times to observe effects similar to those measured with 3R4F AE. This observation is consistent with reduced exposure to harmful and potentially harmful constituents (HPHC) such as carbonyls, whose levels were reduced by 86% on average in THS AE relative to 3R4F AE.

### Nicotine did not promote significant functional or molecular effects in young or old HAoSMCs

**Fig. 6:**
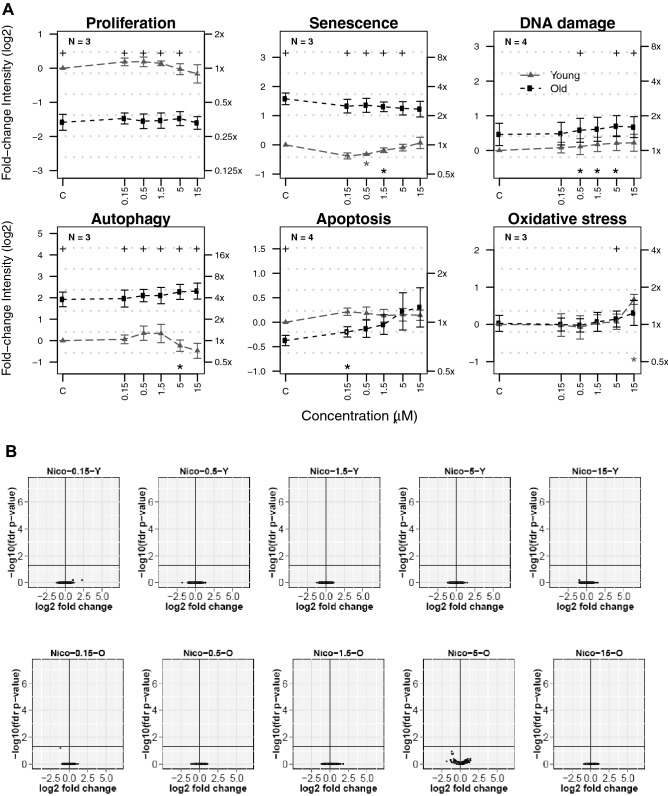
Impact of nicotine on processes involved in cell stress, damage, and fate and gene expression changes in young and old HAoSMCs. **A** The response to various concentrations (log_10_ scale) of aqueous extracts is plotted as the fold change in intensity (log_2_ and original scales on left and right y-axes, respectively) in young (filled gray triangles) and old cells (filled black squares) relative to the control (C) of young cells. Statistical analyses: black crosses at the top of the plot indicate statistical differences in pairwise comparisons between young and old cells at a significance level of 0.05. Stars at the bottom of the plot indicate whether the cell response for each treatment condition is statistically different from its respective control for young (gray stars) and old (black stars) cells separately (*p  <  0.05). Points reflect the mean of *N* independent experiments including n =  2 replicates within each plate. Error bars correspond to their standard error of the mean. **B** SRPs are visualized as volcano plots, with the magnitude of gene expression changes (x-axis) expressed as the fold change in log_2_ scale and the statistical significance (y-axis) represented as − log_10_ (adjusted p value: FDR; *N*  =  3–4 independent experiments). The horizontal line represents the statistical significance threshold (FDR) of 0.05. *C* control; *Nico* nicotine; *O* old; *Y* young

The HCS results show that treatment with nicotine did not affect any of the investigated cellular mechanisms in young or old cells, with the exception of oxidative stress, which was slightly increased in young cells treated with the highest concentration corresponding to 15 µM nicotine (Fig. 6). Apart from this exception, each of the six endpoints in young and old cells remained at levels similar to those in the respective controls up to the highest nicotine concentration tested. Within the range of the tested concentrations, transcriptomics analysis did not reveal significant gene expression changes upon nicotine exposure in young or old HAoSMCs (Fig. 6).

## Discussion

Our study shows that aging HAoSMCs in vitro by increasing the number of cell passages promotes senescence, tends to increase autophagy and DNA damage, and decreases proliferation and apoptosis. These observations are in agreement with previous reports showing that aged human VSMCs obtained by cell culture replication or from old donors (relative to young donors) show increased levels of markers of senescence (β-galactosidase and p16 and p21 oncogenes) and DNA damage (γ-H2AX and DNA strand breaks) and decreased proliferation, as measured by lower bromodeoxyuridine incorporation in old cells (Ruiz-Torres et al. 1999; Nakano-Kurimoto et al. 2009; Guntani et al. 2011; Gorenne et al. 2013; Bielak-Zmijewska et al. 2014; Thompson et al. 2014; Gardner et al. 2015). In the present study, the young and old cells showed no difference in oxidative stress (DHE fluorescence) at baseline; this observation is in contradiction with previous reports showing increased levels of oxidative stress markers in aortic media from old human subjects or in explanted arterial VSMCs from old rats, monkeys, and human subjects relative to samples from young organisms (Fukagawa et al. 2001; Guntani et al. 2011; Csiszar et al. 2012; Miura et al. 2019). Transcriptomics analysis highlighted the impact of aging on the expression of genes involved in the cell cycle (mitosis and DNA repair), matrisome (ECM, collagens, integrins, and focal adhesions), senescence, and marker genes of HAoSMC differentiation (e.g., *ACTA2* and *MYOCD*). These expression changes suggest a modification of the HAoSMC phenotype from contractile to another phenotype, possibly one such as an osteoblatic-like pro-calcificatory phenotype as reported previously (Nakano-Kurimoto et al. 2009; Burton et al. 2010). Additionally, HAoSMC aging increased the expression of genes involved in inflammation/immunity (NFκB signaling, inflammasomes, cytokines, and receptors) and ECM degradation (protease receptor signaling), fibrosis, and autophagy. Measurement of cytokines and proteases in the supernatants of HAoSMCs confirmed that inflammatory mediators, such as CXCL8, IL-6, IL-1β, and IL-1α and proteases, such as MMP-10 [role in atherosclerosis and vascular calcification (Purroy et al. 2018)], were produced and released by HAoSMCs upon aging in vitro. Overall, these results suggest that aging in vitro alters the phenotype and function of VSMCS, which could explain the remodeling and calcification of the arterial vascular wall—a marker of atherosclerosis—in old individuals, aggravated and sustained by an inflammatory microenvironment that favors the recruitment of inflammatory cells in the vascular wall in a feedforward cycle.

For comparative toxicological assessment, AEs from THS aerosol and 3R4F CS were freshly generated and used to expose HAoSMCs. In agreement with previous reports (van der Toorn et al. 2015b; Poussin et al. 2016), the results of chemical analysis clearly showed a reduction in the levels of HPHCs such as carbonyls in THS AE relative to 3R4F AE at an equivalent nicotine concentration. Young and old HAoSMCs were treated with various concentrations of THS AE, 3R4F AE, or nicotine for 24 h. Overall, our results showed that young and old HAoSMCs responded to 3R4F AE in a concentration-dependent manner. However, the magnitude of the response was more pronounced in young HAoSMCs than in old HAoSMCs. At increased concentrations of 3R4F AE, young cells reached similar levels of response to that of old cells, while the baseline response levels of the young and old cells were significantly different for most of the investigated mechanisms, including senescence and proliferation. These observations suggest that exposure to 3R4F AE induces biological effects in young HAoSMCs similar to the aging process observed in old HAoSMCs at baseline. When HAoSMCs are aged, exposure to CS triggers additional injuries promoted by enhanced DNA damage, oxidative stress, apoptosis, and senescence, observations in agreement with previous findings (Chang et al. 2017; Starke et al. 2018). Overall, smoking may accelerate aging by damaging cells and increasing their turnover when cells die. In the context of aged cells/organisms, smoking may exacerbate the processes that were already altered by aging and through crosstalk of activated vascular VSMCs, endothelial cells, and infiltrated immune cells in the vascular wall (Ghosh et al. 2015), leading to vicious cycles and an amplified risk of vascular disorders. Cafueri et al. (2012) have reported that VSMCs from AAA have significantly shorter telomeres, a marker of aging, and increased levels of oxidative DNA damage. Our findings that old HAoSMCs were less responsive to 3R4F AE than young cells could be explained by the fact that the process of aging HAoSMCs had already perturbed many biological mechanisms to levels that closely reached their maximum at baseline and might have reduced the dynamic range of the additional effects triggered by exposure to 3R4F AE.

At 3R4F AE concentrations at which maximum effects were measured in all functional and molecular endpoints in young and old HAoSMCs, THS AE produced no significant effects. It required approximately ten-fold higher concentrations of THS AE to elicit similar effects to those promoted by 3R4F AE in the investigated mechanisms. This observation may be explained by the reduced exposure of HAoSMCs to toxic compounds such as carbonyls, whose levels were shown to be, on average, 86% lower in THS AE relative to 3R4F AE. In addition, our modeling analysis showed that the predicted concentrations of THS AE at which young HAoSMCs reached the marker levels of old HAoSMCs for senescence, autophagy, and apoptosis were 7–21 times higher than the concentrations of 3R4F AE. Overall, these results indicate that, relative to 3R4F AE, THS AE has reduced toxicological effects on HAoSMCs for the investigated pathomechanisms involved in the process of HAoSMC aging and vascular disorders. These findings are consistent with previous in vivo and in vitro reports showing that, relative to 3R4F CS, THS aerosol has a reduced biological impact on the cardiovascular system (van der Toorn et al. 2015b; Poussin et al. 2016; Phillips et al. 2016, 2019; Szostak et al. 2020).

Several studies have assessed the cardiovascular effects of nicotine that translate into increased risk of cardiovascular disease (Li and Dai 2012; Benowitz and Burbank 2016). Some studies have reported the effects of nicotine on vascular cells, such as endothelial cells and VSMCs (Yoshiyama et al. 2014; Rabkin 2016; Kugo et al. 2017; Li et al. 2018; Wagenhauser et al. 2018; Wu et al. 2018; Wang et al. 2019). In human VSMCs, nicotine was shown, for example, to induce the production of MMP2, a metalloproteinase involved in the development of AAA (Wang et al. 2012). Other in vivo and in vitro studies have reported that nicotine modulates the migration and proliferation capacities of rodent VSMCs through phenotype switching (Ren et al. 2018; Wang et al. 2019). In the present study, findings related to the investigated mechanisms using HCS as well as transcriptomics and MAP analysis did not reveal significant effects of exposure of HAoSMCs to nicotine. A slight increase in oxidative stress was observed in young HAoSMCs at the highest concentration of nicotine (15 μM). The range of nicotine concentrations tested (0.15–15 μM) spanned the physiological concentrations of nicotine measured in the plasma of smokers (0.07–0.23 μM; Russell et al. 1981; D’Ruiz et al. 2015; Brossard et al. 2017). In many pre-clinical studies, the nicotine concentrations used are  ≥  10 μM, which could be considered to be supraphysiological, given that the plasma levels of nicotine after smoking of a cigarette peak at 15–20 ng/mL, corresponding to approximately 0.13 μM (Benowitz et al. 2006, 2009). Studies on nicotine medications and smokeless tobacco indicate that the risks of nicotine use without tobacco combustion products such as CS are low compared with the risks of cigarette smoking (Benowitz and Burbank 2016).

In summary, this study shows that the procedure of aging HAoSMCs in vitro triggers molecular changes that may alter the phenotype and function of the HAoSMCs and increase the risk of vascular disorders. In young HAoSMCs, exposure to CS AE triggers similar changes to those observed at the end of the aging process in vitro; in old HAoSMCs, it exacerbates the impact of aging with additional effects. Relative to 3R4F AE, THS AE showed a significantly reduced biological impact in young and old HAoSMCs, demonstrating the potential of THS to reduce smoking-dependent vascular aging and activation of pathomechanisms leading to cardiovascular disorders in comparison with cigarettes.

## Supplementary Information

Below is the link to the electronic supplementary material.

Supplementary file 1 (PDF 927 KB)

Supplementary file 2 (XLSX 333 KB)

Supplementary file 3 (XLS 178 KB)

## Data Availability

Datasets, further detail on the protocols, and additional data visualizations are available on the INTERVALS platform at https://doi.org/10.26126/intervals.x3xkne.1.
