# Supplementary material for: Systems toxicology study reveals reduced impact of heated tobacco product aerosol extract relative to cigarette smoke on premature aging and exacerbation effects in aged aortic cells in vitro
Source: Arch Toxicol. 2021 Jul 27;95(10):3341–59. doi: 10.1007/s00204-021-03123-y (PMC8448694; doi:10.1007/s00204-021-03123-y)
Supplement: Supplementary file 1 — (PDF 927 KB) [file 204_2021_3123_MOESM1_ESM.pdf]

Systems toxicology study reveals reduced impact of heated tobacco product aerosol extract relative to cigarette smoke on premature aging and exacerbation effects in aged aortic cells in vitro

Archives of Toxicology

Carine Poussin[1]*, Marco van der Toorn[1]*, Sophie Scheuner[1], Romain Piault[1], Athanasios Kondylis[1], Rebecca Savioz[3], Rémi Dulize[1], Dariusz Peric[1], Emmanuel Guedj[1], Fabio Maranzano[1], Celine Merg[1], Moran Morelli[1], Anne-Laure Egesipe[1], Stéphanie Johne[1], Shoaib Majeed[1], Claudius Pak[1], Thomas Schneider[1], Walter K. Schlage[2], Nikolai V. Ivanov[1], Manuel C. Peitsch[1], Julia Hoeng[1]

*Equal contribution

[1]PMI R&D, Philip Morris Products S.A., Quai Jeanrenaud 5, CH-2000 Neuchâtel, Switzerland
[2]Biology Consultant, Max-Baermann-Str. 21, 51429 Bergisch Gladbach, Germany
[3]Consultants in Science Sàrl, Biopole, Route de la Corniche 4, 1066 Epalinges, Switzerland

*Corresponding author:
Dr. Carine Poussin
PMI R&D, Philip Morris Products S.A.
Quai Jeanrenaud 5
2000 Neuchâtel
Switzerland
Tel: +41 58 242 2119
Fax: +41 58 242 2811
Email: Carine.Poussin@pmi.com

Online resource 1: Supplementary materials and methods

Network perturbation amplitude analysis

By using a newly developed computational approach, network perturbation analysis was performed to quantify the response of HAoSMCs to aging, product exposure, and the combination of aging and product exposure (Martin et al. 2014). This analysis leveraged biological cause-and-effect network models as prior knowledge as well as systems response profile (SRP) data corresponding to pairwise comparisons at the gene level of two conditions (e.g., control vs treatment). A network is an assembly of directed and signed causal relationships between molecular biological entities (e.g., protein abundance and activities, chemical compounds, biological activity, and gene expression) that collectively models a specific biological process, such as cell proliferation, inflammation, or apoptosis, in a defined context (e.g., lung or vascular). The causal relationships have been curated from the scientific literature manually and encoded in Biological Expression Language syntax. The network is composed of functional (backbone) and transcript (gene expression) layers. The backbone node-level and network-level perturbation amplitudes can be calculated by considering measured stimulus vs. control gene expression changes corresponding to the so-called SRP (transcript) and the network topology (backbone layer) by using a backward-causal reasoning algorithm. Three statistics are computed to assess the significance of a network perturbation amplitude (NPA) score with respect to biological variation (confidence interval) and its specificity to the given two-layer network structure (O and K statistics). A collection of biological networks representative of main processes representative of cell fate (CFA), cell proliferation (CPR), cell stress (CST), inflammation (inflammatory process network, IPN), tissue repair and angiogenesis (TRA), and vascular inflammation (vascular inflammatory processes, VIP) was used to analyze the HAoSMC (6 networks comprising 52 subnetworks) cell transcriptomics data (all networks have been published and are accessible at http://causalbionet.com (Boue et al. 2015; sbv IMPROVER project team et al. 2013)).

Online resource 2 Principal component analyses for identifying main sources of variations and genes that drive these effects ("driver genes")

PCA Name	SRPs for PCA	Nb SRPs	%Variance-Top3 PC	%Variance	Prototype Gene Plus	Prototype Gene Minus	Highlighted Effect
**pca.3R4F.THS.C-OvsY** (Comparison of all conditions with those in young cell control)	C-Oy.a/3R4F-0.0275-Y/3R4F-0.055-Y/3R4F-0.11-Y/3R4F-0.22-Y/3R4F-0.0275-Oy/3R4F-0.055-Oy/3R4F-0.11-Oy/3R4F-0.22-Oy/C-Oy.b/THS 2.2-0.22-Oy/THS 2.2-0.22-Y/THS 2.2-0.44-Y/THS 2.2-0.88-Y/THS 2.2-1.76-Y/THS 2.2-0.22-Oy/THS 2.2-0.44-Oy/THS 2.2-0.88-Oy/THS 2.2-1.76-Oy/	18	81.14	PC1 = 47.94	CXCL12	CXCL6	Cell aging
				PC2 = 22.03	HSPA6	GIMAP7	High and low product concentrations
**pca.3R4F.THS.vsY.vsO** (Comparison of old and young cell conditions with those in old and young cell controls, respectively)	3R4F-0.0275-Y/3R4F-0.055-Y/3R4F-0.11-Y/3R4F-0.22-Y/3R4F-0.0275-O/3R4F-0.055-O/3R4F-0.11-O/3R4F-0.22-O/THS 2.2-0.22-Y/THS 2.2-0.44-Y/THS 2.2-0.88-Y/THS 2.2-1.76-Y/THS 2.2-0.22-O/THS 2.2-0.44-O/THS 2.2-0.88-O/THS 2.2-1.76-O/	16	64.84	PC1 = 45.9	HSPA6	GIMAP7	High and low product concentrations
				PC2 = 10.3	PRSS35	CYP1A1	Concentration-dependent product effect
**pca.3R4F.THS.vsY.vsO.Low** (Comparison of old and young cell conditions with those in old and young cell controls, respectively — Low product concentrations contrasts only)	3R4F-0.0275-Y/3R4F-0.055-Y/3R4F-0.11-Y/3R4F-0.0275-O/3R4F-0.055-O/3R4F-0.11-O/THS 2.2-0.22-Y/THS 2.2-0.44-Y/THS 2.2-0.88-Y/THS 2.2-0.22-O/THS 2.2-0.44-O/THS 2.2-0.88-O/	12	53.43	PC1 = 26.0	HIST1H1B	CYP1A1	Concentration-dependent product effect
				PC2 = 15.8	CCL8	HTATSF1P2	3R4F and THS 2.2 experiments

PCA Name	SRPs for PCA	Nb SRPs	%Variance-Top3 PC	%Variance	Prototype Gene Plus	Prototype Gene Minus	Highlighted Effect
**pca.3R4F.vsY.vsO** (Comparison of old and young cell conditions with those in old and young cell controls, respectively — 3R4F contrasts only)	3R4F-0.0275-Y/3R4F-0.055-Y/3R4F-0.11-Y/3R4F-0.22-Y/3R4F-0.0275-O/3R4F-0.055-O/3R4F-0.11-O/3R4F-0.22-O/	8	77.66	PC1 = 42.4	HSPA6	GIMAP7	High and low product concentrations
				PC2 = 22.5	CYP1A1	EML6	Concentration-dependent product effect
**pca.THS.vsY.vsO** (Comparison of old and young cell conditions with those in old and young cell controls, respectively — THS contrasts only)	THS 2.2-0.22-Y/THS 2.2-0.44-Y/THS 2.2-0.88-Y/THS 2.2-1.76-Y/THS 2.2-0.22-O/THS 2.2-0.44-O/THS 2.2-0.88-O/THS 2.2-1.76-O/	8	83.38	PC1 = 63.3	HSPA6	GIMAP7	High and Low product concentrations
				PC2 = 12.8	ANXA10	PRSS35	Combined cell aging and concentration-dependent product effect
**pca.3R4F.THS.vsY.Yonly** (Comparison of young cell conditions with those in young cell controls — Young cell contrasts only)	3R4F-0.0275-Y/3R4F-0.055-Y/3R4F-0.11-Y/3R4F-0.22-Y/THS 2.2-0.22-Y/THS 2.2-0.44-Y/THS 2.2-0.88-Y/THS 2.2-1.76-Y/	8	83.24	PC1 = 57.8	HSPA6	TNFSF10	High and low product concentrations
				PC2 = 15.1	FAM227B	EML6	3R4F and THS experiments
**pca.3R4F.THS.vsO.Oonly** (Comparison of old cell conditions with those in old cell controls — Old cell contrasts only)	3R4F-0.0275-O/3R4F-0.055-O/3R4F-0.11-O/3R4F-0.22-O/THS 2.2-0.22-O/THS 2.2-0.44-O/THS 2.2-0.88-O/THS 2.2-1.76-O/	8	79.7	PC1 = 54.6	HSPA6	GIMAP7	High and low product concentrations
				PC2 = 16.6	HTATSF1P2	SELE	3R4F and THS experiments

"Prototype Gene Plus/Minus" means that the projection value of the gene vector on PCx is positive/negative.

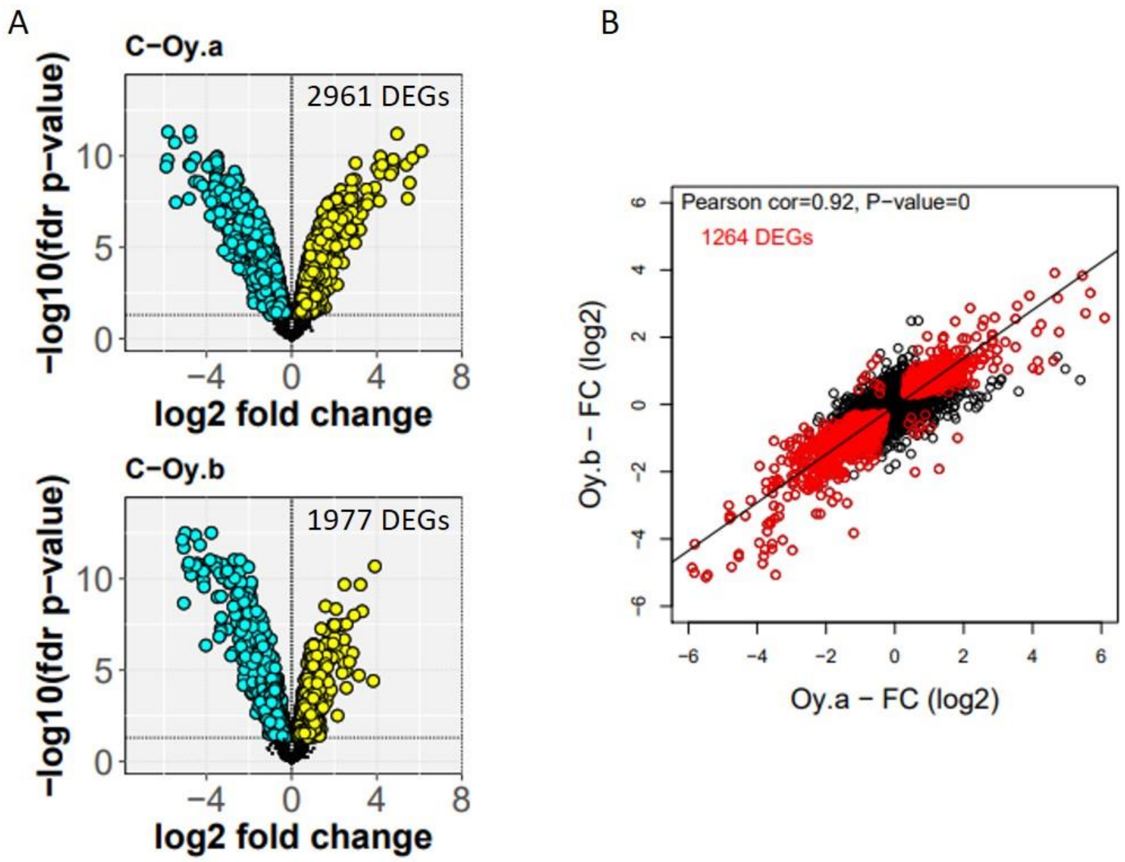

Online resource 3 Comparison of gene expression changes between old and young HAoSMCs at baseline. (A) Gene expression differences between old and young HAoSMCs at baseline are visualized as volcano plots, with the magnitude of gene expression changes (x-axis) expressed as the fold change (FC) in log2 scale and the statistical significance (y-axis) represented as -log10 (adjusted p value: FDR) (N = 4 independent experiments for C-Oy.a and C-Oy.b). The horizontal line represents the statistical significance threshold (FDR) of 0.05. FC plot between OvY SRPs for experiments A and B, conducted independently (different plates) and corresponding to the experiments performed with 3R4F and THS AEs, respectively. (B) The Pearson correlation coefficient was calculated between both SRPs for commonly differentially expressed genes highlighted in red. The number of differentially expressed genes (DEG) are indicated in the top right corner of the plots.

Online resource 4 Pathways and disease gene set enrichment analysis using MSigDB-C2CP and Qiagen gene set collections and associated with up- and downregulated gene expression changes in old versus young HAoSMCs.

Filename: Supplementary file 2.xlsx

Online resource 5 Concentrations of nicotine and eight carbonyls in aqueous extracts generated from THS aerosol and 3R4F smoke

Compound (µg/item trapped)		Nicotine	Formaldehyde	Acetaldehyde	Acetone	Acrolein	Propionaldehyde	Crotonaldehyde	Methyl ethyl ketone	Butyraldehyde
**3R4F**										
	Mean	71.8	52.1	1118.7	552.2	66.9	62.8	34.8	123.3	17.3
	SD	8.6	6.9	75.4	84.7	10.4	8.6	3.5	38.8	2.5
**THS**										
	Mean	69.9	3.5	166.2	36.5	3.2	11.4	1.2	4.3	7.0
	SD	18.9	1.1	15.3	12.6	0.5	0.8	0.2	1.8	0.7
**% Reduction THS vs. 3R4F**		-	93.4*	85.1*	93.4*	95.2*	81.8*	96.5*	96.5*	59.5*

**Statistically significant differences (Welch t-test; $p < 0.05$) between 3R4F and THS. N = 11 and 9 independent generations of aqueous extracts from 3R4F cigarette smoke and THS aerosol, respectively. Bars are means ± standard deviation.*

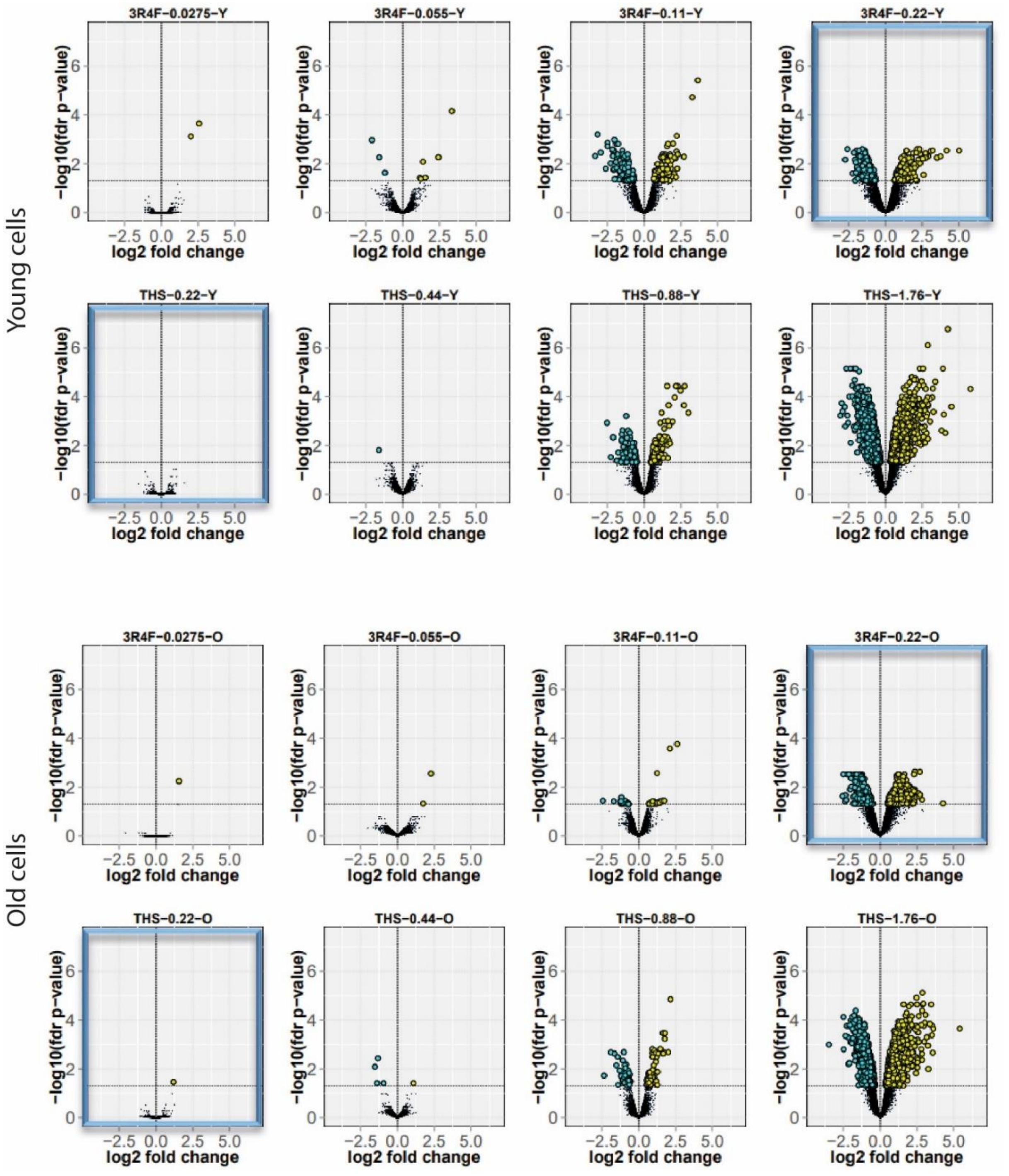

Online resource 6. Concentration-dependent effects of 3R4F and THS AE on gene expression changes in young and old HAoSMCs. Gene expression differences between each 3R4F/THS AE concentration in young and old HAoSMCs with its vehicle control in young and old HAoSMCs, respectively, are visualized as volcano plots, with the magnitude of gene expression changes (x-axis) expressed as the fold change (FC) in log2 scale and the statistical significance (y-axis) represented as -log10 (adjusted p value: FDR) (N = 4 independent experiments). The horizontal line represents the statistical significance threshold (FDR) of 0.05. The blue square outlines the bridging AE concentration between 3R4F and THS.

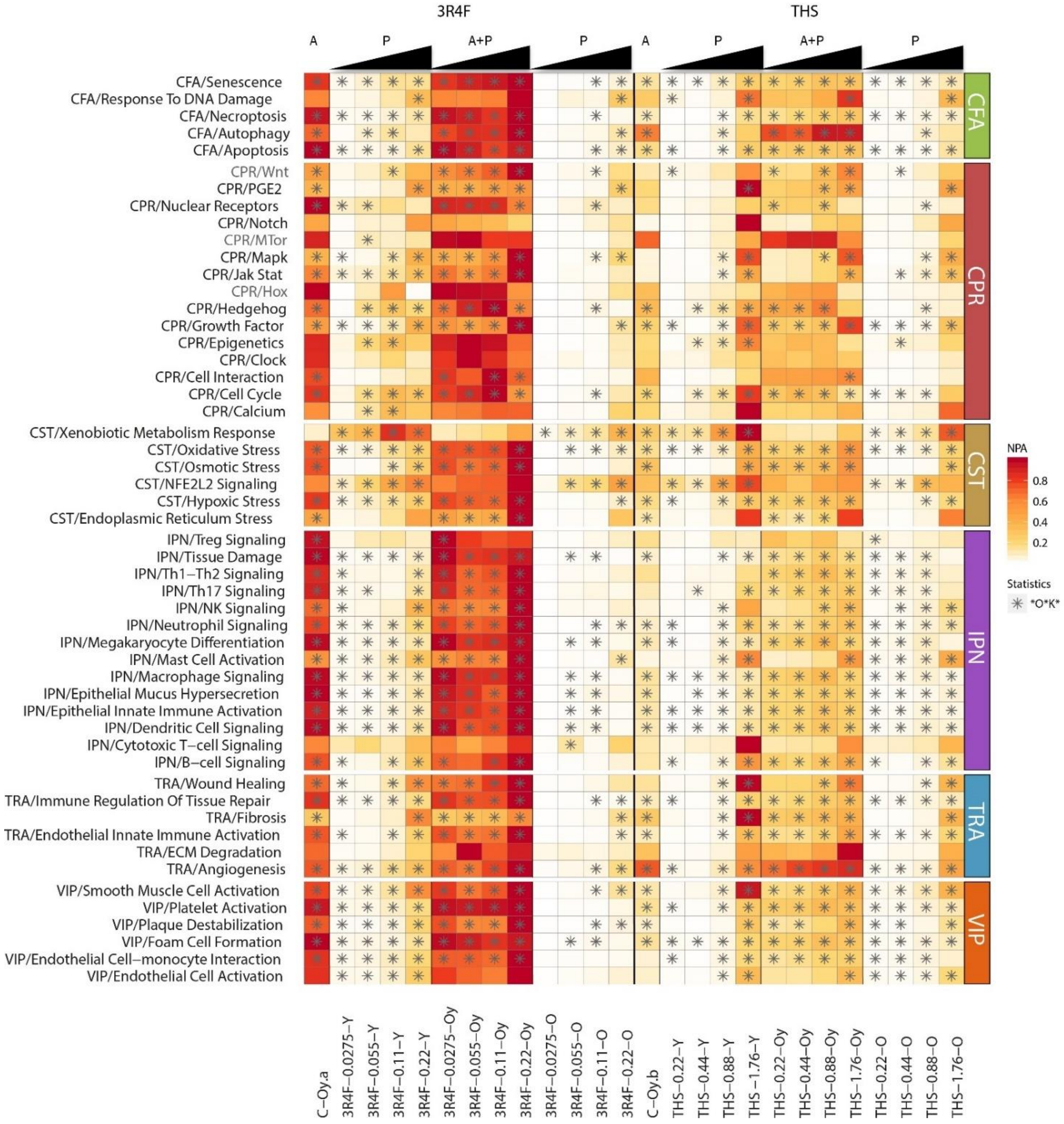

Online resource 7 Heatmap of network perturbation amplitudes for the effects of THS and 3R4F AE exposure. Heatmap of NPA scores computed with HAoSMC systems response profiles for 52 subnetworks. Black stars indicate that the NPA score is significant with regard to experimental variation and O and K statistics with $p < 0.05$. Subnetworks are grouped by networks representative of higher-level biological processes. "A" represents the aging effect — the responses of old cells were compared with those of young cells. "P" represents the product effect — the responses of young (Y) and old cells (O) were separately compared with the responses of their respective controls. "A+P" represents the combined effect of aging and product — the responses of old cells were compared with those of the young cell control (Oy). Abbreviations: A, aging; P, product; CFA, cell fate; CPR, cell proliferation; CST, cell stress; TRA, tissue repair and angiogenesis; IPN, inflammatory process networks; VIP, vascular inflammatory processes; NPA, network perturbation amplitude.

Online resource 8 Complete results of pathways and biological processes overrepresented in the list of genes correlated with the expression patterns of the driver genes shown in Table 1.
Filename: Supplementary file 3.xls
